# Boar management and semen handling factors affect the quality of boar extended semen

**DOI:** 10.1186/s40813-017-0062-5

**Published:** 2017-07-25

**Authors:** Alfonso Lopez Rodriguez, Ann Van Soom, Ioannis Arsenakis, Dominiek Maes

**Affiliations:** Department of Reproduction Obstetrics and Herd Health, Faculty of Veterinary Medicine, Ghent University, Salisburylaan 133, 9820 Merelbeke, Belgium

**Keywords:** Artificial insemination, Bacteriospermia, Boar, Breeding, Management, Semen collection, Semen quality

## Abstract

Artificial insemination (AI) is the preferred method for reproduction in the majority of the intensive pig production systems Worldwide. To this end, fresh extended ready-to-use semen doses are either purchased from AI-centres or produced by boars kept on-farm. For profitable semen production, it is necessary to obtain a maximum amount of high quality semen from each boar. This paper reviews current knowledge on factors that may affect semen quality by influencing the boar or the semen during processing.

Genetic markers could be used for early detection of boars with the highest fertility potential. Genetic selection for fast growth might jeopardize semen quality. Early detection of boars no longer fit for semen production might be possible by ultrasonography of the testes. Seasonal variation in sperm quality could be associated with changes in photoperiod and heat stress during summer. Comfortable housing, with appropiate bedding material to avoid locomotion problems is essential. In some areas, cooling systems may be necessary to avoid heat stress. The sperm quality can be manipulated by feeding strategies aiming, for instance, to increase sperm resistance to oxidative stress and extend storage duration.

High collection frequency will negatively influence sperm quality. Also, if collection is not hygienically performed it will result in bacterial contamination of the semen doses. The concern over bacterial contamination has risen not only because of its negative effect on semen quality but also due to the detection of antimicrobial resistance in isolates from extended semen. Moreover, bacterial and viral pathogens must be monitored because they affect semen production and quality and constitute a risk of herd infection. During processing, boar sperm are submitted to many stress factors that can cause oxidative stress and capacitation-like changes potentially reducing their fertility potential. Dilution rate or dilution temperature affects the quality of the semen doses. Some packaging might preserve semen better than others and some plastic components might be toxic for sperm. Standard operation procedures and quality assurance systems in AI centres are needed.

## Background

During the last decades, the use of porcine semen for artificial insemination (AI) by means of fresh diluted semen has increased considerably [1, 2]. Compared to natural mating, AI reduces the risk of disease transmission [3, 4], it allows the introduction of superior genes into sow herds and additionally, it leads to a better profitability of each boar ejaculate. Therefore, AI has become a very useful tool in countries with intensive pig production. In Western Europe, more than 90% of the sows have been bred by AI for more than two decades [1, 2]. Semen is obtained from boars present either in the sow farm or in specialised AI-centres. The latter offer a diversity of breeds and genetic lines and the potential of distributing ready-to-use semen doses of constant quality. To obtain the maximum productivity from each boar, genetic and external factors that might affect semen quality and production must be taken into consideration.

Selecting boars with the best semen quality at an early age is imperative to reduce the costs of raising animals that will not be used for semen production [5]. Once in production, boars that are delivering semen of poor quality are normally put on rest for few weeks and are culled if they do not recover. This implies extra costs for AI centers and they would therefore benefit from a method for early detection of boars that must be culled [6]. Some morphological markers could be used to identify boars from an early age that will produce high-quality semen and also could be used to facilitate genetic improvement by utilizing those phenotypic differences in order to select the most superior genotypes [7]. In addition, not only semen production parameters have to be considered when selecting boars for AI, but also their progeny’s potential for meat production [8].

Housing, feeding and management may have a direct impact on semen output [9–11] and therefore on the profitability of an AI centre. During processing and storage, sperm suffer stress and it is believed that some feeding strategies can prepare the semen to better resist this stress [12].

In addition to boar management, ejaculates need to be handled in a controlled manner to avoid sperm damage. An increasing number of AI centres are implementing quality control systems in which every step from collection to packaging is monitored [13]. The temperature of dilution and storage or the different compositions of the extenders are known to influence semen quality [14, 15]. Bacterial contamination has been identified as one of the most critical problems during fresh semen processing [13]. Not only the negative effect of bacteria on semen quality is of concern but also the increase in bacteria isolated from extended semen showing resistance against antibiotics [16].

The present paper will review and critically discuss the different steps during the production of extended pig semen. Emphasis will be put on factors that could jeopardize semen quality such as boar management, bacterial contamination, semen collection, processing and storage.

## Managing the boar: Factors affecting semen production and quality

Important management factors of the boar affecting semen production and quality are summarised in Table 1.

**Table 1:** Factors related to boar management affecting boar semen quality

Factor	Details	Effect of semen quality	Reference
Boar selection			
Breed	Pietrain and Duroc vs. GL, LW, YS	Reduced sperm counts	[5]
Back fat	Back fat thickness in highly selected Piétrain	Positive association with motility	[8]
Housing			
Group housing	Groups of prepupebertal boars vs individual	Higher sperm counts	[45]
Photoperiod	24 h of complete light or darkness for 3 months	Reduced volume and concentration	[11]
Heat stress	34.5 °C (8 h) or 31 °C (16 h) for 90 days vs. 23 °C	Reduced motility, reduced morphology	[48]
Nutrition			
Feed restriction	1.4 times below maintenance	Reduced sperm counts	[51]
Protein restriction	12.0% crude protein (CP) in growers	Reduced sperm counts	[51]
Selenium	0.06 ppm vs 0.5 ppm continuously	Reduced motility, reduced morphology	[56]
	Organic vs inorganic	Increase concentration, increase oxidative stress, increase PGHX	[57, 60]
L carnitine	625 mg/boar/day supplementation	Improved morphology in Piétrain	[61]
Collection			
Collection frequency	Twice a day during 4 days compared to once every 2 days	Reduced motility and morphology	[9]
Collection pen	Pen allowing sexual stimulus	higher sperm counts	[63]

*GL* German Landrace, *LW* Large white, *YS* Yorkshire

### Genetics and boar selection

Appropriate boar selection is crucial and only boars with the best traits for sperm production should be retained. If selection is based on semen quality, it must be taken into account that the sperm quality of boars younger than 8 months is lower than in older boars [5]. Therefore, selecting boars for AI at young ages based on semen quality can be misleading. Having to wait 8 months for selection has the disadvantage that it slows down the speed of genetic improvement. Also, boars culled for poor semen quality will have developed boar taint unless castrated, leading to a reduced price when slaughtering. It has been proposed that boars could be vaccinated against gonadotropin-releasing hormone (GnRH), with a single injection at 16 weeks without this having a negative effect on further boar semen quality [17]. However, this strategy should be implemented with care because this vaccination when applied twice reduces testicle size and could have a negative effect on boar semen.

Birth weight has been proposed as a criterion to select boars with the highest potential at an early age [18, 19]. It seems that post-pubertal boars have smaller testes size when they had a low birth weight (around 1 kg) compared to boars with high birth weight (around 2 kg). However, semen quality (motility, volume, concentration, DNA and acrosome integrity and sperm morphology) does not seem to be affected by birth weight. Selection of boars can be done at an older age (150 days) based on the testicular size, that has been shown to be positively associated with total sperm per ejaculate, although there was no association with motility, volume or morphology [20].

For obvious reasons, the customer demands semen from genetic lines that will result in offspring with good potential for meat production, thus with good growth, feed efficiency and lean carcass composition. It has been suggested that selection of boars to improve growth rate may have a negative effect on semen quality as was described in other species such as chicken or the double-muscled Belgian Blue beef breed [21, 22]. Recently, it has also been suggested that back fat thickness in highly selected Piétrain boars is positively associated with progressive motility [8]. Interestingly, in the latter study, the association of back fat with total sperm motility seemed to be dependent on storage duration of extended semen. From day 0 to day 2 of storage boars with higher back fat had higher sperm motility, while the association was negative from day 3 to day 4, suggesting a negative association of back fat with semen resistance to storage. It is possible that these phenotypic correlations are the result of indirect selection and interactions between several genes. It has also been shown that boars of lines selected for ovulation rate and uterine capacity produce more sperm compared to non-selected lines [23]. Also, it seems that Piétrain and Duroc boars have lower sperm counts than German landrace, Large white and Yorkshire boars [5]. In the same study, some differences were observed between breeds for motility and morphology, but the differences were small. It seems more likely that individual variation will exceed breed variation.

Apart from semen production and quality, it has been proposed that genetic markers should be investigated before introducing boars into production. Such markers could pertain to young age at sexual maturity, higher resistance to heat stress or better maintenance of semen quality during liquid or frozen storage [7]. For instance, sperm abnormalities in Finnish Yorkshire boars such as the knobbed acrosome, spermatogenic arrest, or sperm immotile short tail defect seem to have a genetic origin. Some candidate genes have been proposed as the cause, suggesting that genetic selection of the boars could avoid having these defects in the sperm of the progeny [24–26]. Recently, the mitochondrial methionyl-tRNA formyltransferase gene has been proposed as a marker associated with sperm motility [27].

### Genital pathologies and infectious pathogens affecting boar health

A slaughterhouse investigation of boars culled due to fertility problems revealed different testicular pathologies such as varicocele, fibrosis, inflammation or haemorrhages, with varicocele being the most common finding [28]. Also, some tumours (e.g. haemangioma or Sertoli cell tumour) have been described in boars and may lead to poor semen quality [29]. Boar AI centres would benefit from a method for early detection of these conditions, e.g. by ultrasound examination of the scrotum and testicles [6]. Semen of unilateral cryptorchidic boars should not be used because the quality as well as the production of the semen in the scrotal testicle will be impaired [30]. Epididymis dysfunction seems to lead to a high incidence of sperm with single bent tails and low motility [31].

Infectious diseases may also jeopardize semen quality. *Brucella suis,* leptospires and *Chlamydia sp.* [32, 33] are bacteria known to negatively affect semen quality. In tropical climates, trypanosomes [34] can disrupt spermatogenesis. Several viral agents can also affect semen quality and production as recently reviewed [4]. Japanese encephalitis virus causes orchitis that results in reduced sperm counts and motility and increases sperm abnormalities [35]. Aujezsky’s disease virus causes testicular degenerations and increases sperm abnormalities [36]. Porcine reproductive and respiratory syndrome virus (PRRSv) does not cause specific lesions in the testes but infection with the virus may result in sperm abnormalities, reduced motility and lower ejaculate volume due to a direct influence of the viral replication in the spermatogenic epithelium [37, 38]. Schulz et al. [37] found that some computer assisted semen analysis (CASA) parameters (increased ALH and reduced linearity) changed after PRRSv infection and proposed that monitoring these parameters routinely could be used as a first indication of a PRRSv outbreak [37] Epididymitis, orchitis and permanent or temporary infertility, increased abnormalities or azoospermia have been observed after artificial infection of boars with Rubulavirus (blue eye disease) [39]. Porcine enteric picornavirus infection may result in seminal vesiculitis, decreased libido and more sperm abnormalities. Very recently the effect of intestinal parasites on semen quality has been investigated. A significant association between parasitic infection measured as eggs/g faeces and semen quality was not demonstrated [40]. In the latter study, there seemed to be an effect of the deworming regimen on total sperm cells. The highest sperm counts were found in those AI centres that did not treat for intestinal worms compared to AI centres that dewormed either only in the quarantine or both in the quarantine and during production with macrocyclic lactones or benzimidazoles in different combinations. These findings are difficult to evaluate because many different variables were included in the statistical models of this epidemiological study. A randomized controlled study testing the effect of different deworming products on semen quality would give clearer results.

Vaccination for some diseases may protect the boars from infection and control or prevent transmission. Vaccination against parvovirus, PRRSv or porcine circovirus type 2 may help to reduce shedding of the virus following infection [41, 42]. Apart from their effect on virus shedding, the effect of different vaccines on semen quality has not been investigated in detail. The sperm of boars vaccinated with modified live vaccine against PRRSv and subsequently challenged with the virus had worse motility and morphology than of boars challenged but not vaccinated [41]. As mentioned earlier in this section, PRRSv replicates in spermatogenic epithelium [38] and it is therefore possible that the vaccine’s live virus may have a similar effect. Modified live PRRSv vaccines in Eurpope are registered for use in piglets and sows only, and therefore their effect on semen quality has not been investigated in detail. More recently, vaccination against PCV2 with an oil-based adjuvant vaccine has been reported to cause fever and lethargy that could lead to reduced semen quality [43]. However, this adverse event was only observed in one out of four boars which makes it difficult to draw a conclusion about the safety of oil-based vaccines for use in boars.

### Boar housing and stable climate conditions

According to European legislation (Commission directive 2001/93/EC), at least 6 m^2^ of solid floor area have to be available per boar; the housing must allow the boar to turn around and to hear, smell and see other boars. The type of housing for mature boars may affect boar health and indirectly affect semen quality by bacterial contamination [44]. When bedding is supplied, remainders of bedding on the ventral abdomen of the boar should be removed before semen collection to avoid bacterial contamination of the ejaculate [44].

The housing of young boars also influences semen production. It has been shown that group housing of growing boars is beneficial for subsequent reproductive performance. Groups of 8 boars housed in pens of 4 m × 4.3 m from 30 kg until they successfully completed two mountings (approx. 6 months of age), had on average stronger legs for mounting, higher libido, earlier accomplishment of the first mating and higher sperm counts compared to boars housed individually [45].

Besides an appropriate pen construction, the environment must also be adapted to the boars’ requirements. The effect of different light regimes, temperatures, humidities and seasonal effects on semen quality have been investigated in different studies. The role of light regime on semen quality is controversial. Boars kept under natural light plus artificial light supplementation (10–500 lx) to maintain constantly 15 h of light/day from 11 weeks of age until puberty (24–26 weeks), had a faster sexual maturation and a higher libido than boars receiving only natural light during that period (15 h at 11 weeks to 9 h at the end of the trial) [46]. However, there was no effect maintaining a constant 15 h light on semen quality. Length of light exposure seems to have an effect on semen quality in extreme conditions, as showed in a more recent study where adult boars were submitted to either 24 h of artificial light or 24 h of complete darkness for a period of 3 months. These extreme regimes had a negative effect on semen volume and concentration, especially when boars were submitted to complete darkness compared to 12 h [11]. In the latter study, there was a reduction in semen volume and concentration after 1 month of exposure to 24 h of light or of complete darkness but after 3 months semen volume and concentration returned to the values before treatment. The authors suggested that boars were able to adapt to these extreme photoperiods. However, similar to other studies, photoperiod did not have an effect sperm motility or vitality [46, 47].

Heat stress also influences boar semen quality. Boars exposed to 34.5 °C for 8 h and 31.0 °C for 16 h daily for 90 days had lower sperm motility and sperm morphology as well as reduced fertility compared to control boars maintained at 23.0 °C [48]. It seems also that maternal purebred lines are more sensitive to warmer temperatures [49]. Moreover, not only constant heat stress but also fluctuations of more than 10 °C (25–35 °C) in temperature between the day and the night and a humidity over 90% may decrease sperm production [50].

To our knowledge there is no research on the influence of air quality, air filtration, ammonia or other gas concentrations on semen quality of boars or other species, but it seems obvious that a good air quality is imperative for the comfort and welfare of the boar.

### Boar nutrition

A review of the nutritional requirements for boars concluded that only severe feed restriction, i.e. feed levels below 1.4 times maintenance, have a negative effect on sperm output and/or libido but it does not seem to affect sperm motility or vitality [51].

As for feed and energy intake, it seems that only severe deficiencies in protein in the diet will affect boar libido and sperm output but with no effect on semen quality [51]. The appropriate protein level in feed for boars in production is controversial and based on older data. More recently, it has been shown that, when boars are fed diets with a low protein level (13%), increasing threonine:tryptophan:arginine ratio of the protein content will enhance boar semen quality [10].

Much research has focussed on different feed supplementations in boar feed. Special attention has been paid to antioxidants since it is believed that one major cause of sperm damage during liquid storage is peroxidation of the sperm membrane lipids [52]. Different studies have shown that sperm lipid composition can be modified by feed supplementation with n-3 polyunsaturated fatty acids (PUFA) [12, 53]. However, whether feed supplementation with PUFA has an effect on sperm resistance to storage is controversial. In one study, tuna oil supplementation (30 g tuna oil/kg diet) during 6 weeks improved sperm motility, acrosome integrity and morphology [53]. In contrast, no effect was found of supplementation with tuna oil (60 g/boar/day) during 6 months on sperm viability, motility, acrosomal integrity, susceptibility to peroxidation, and DNA fragmentation or on semen quantity compared to supplementation with hydrogenated animal fat (62 g/boar/day) or menhaden oil (60 g/boar/day) [12]. Selenium (Se) has received much attention for its antioxidant properties as a structural component of glutathione peroxidase (GPx), an enzyme present in boar sperm which protects cellular and subcellular membranes against peroxidation [54, 55]. In boars, supplementation of a basal diet containing 0.06 ppm Se with 0.5 ppm Se from weaning to 9 months of age resulted in higher sperm motility and less abnormal sperm than in the boars fed the non-supplemented basal diet [56]. In the latter study, higher fertility rates were observed in gilts inseminated with semen from the boars fed the Se-supplemented diet. Also, the form (inorganic or organic) in which Se is given to the boars may have an effect on semen quality although results are contradictory. We showed that changing from inorganic to organic Se in the diet of adult boars increased sperm concentration but reduced straight forward motility and resistance to oxidative stress [57]. Subsequent studies did not find differences in semen quality of boars fed organic or inorganic Se [58, 59]. Also recently, more GPx was found in organic Se fed boars but no effect on semen quality was observed [60].

The association between vitamins such as L-carnitine or Vit E and semen quality has been investigated. Supplementation with L-carnitine (625 mg/boar/day) enhanced the number of mature sperm in ejaculates from Piétrain boars when photoperiod and temperature increased, but this beneficial effect was not observed in Duroc and Large White boars [61]. Vitamin E works together with Se to protect sperm against lipid peroxidation and deficiencies in this vitamin in feed may result in reduced motility and more abnormal sperm [56]. In another study, supplementation with a mix of different fat and water soluble vitamins did not reduce the negative effect of high collection frequencies on sperm production or quality [62].

### Collection pen and collection frequency

Similar to the housing pen, the collection pen must be safe for boars and employees and should allow fast processing of many boars [63]. Automation of the collection line allowing for almost hands free collection has been recently developed [64]. This system includes, among other features, pneumatic opening of access doors and electronic identification of collector and boar and it has been proven to increase the number of boars processed per collector per hour without decreasing sperm production (concentration and volume). Different manufacturers of automated semen collection systems claim to reduce bacterial contamination. Whether these automated systems have an effect on semen quality, hygiene or contamination of the ejaculate, deserves further investigation.

The design of the collection pen also influences boar sexual behaviour [63]. Boars that have a sexual stimulus seem to complete collection faster thus resulting in more boars processed in a shorter period of time. Additionally, these boars seem to have higher sperm counts [63, 65]. Boars can be sexually stimulated just before collection by allowing them to see other boars in action with the dummy. This is possible when a so-called warm up area is available prior to the collection pen. Boar stimulation with prostaglandins (PGF2α) has also been studied but, apart from a tendency to a reduced time to onset of ejaculation and a longer duration of the ejaculation, no effect was observed on sperm counts or semen quality [66].

Lack of hygiene during collection will result in bacterial and viral contamination of the ejaculate and subsequently of the semen doses [44, 67, 68]. Additionally, the hair surrounding the preputial orifice must be trimmed on a regular basis because it could result in bacterial contamination [44]. Bacterial contamination may cause a decrease in semen quality by direct effects of bacteria or by indirect action of bacterial by-products on sperm [69, 70].

Generally, semen from boars in AI-centres is collected approximately twice per week [1]. It is known that a high frequency of collection has a negative effect on sperm morphology and motility because sperm is forced to rapidly pass from caput to cauda of the epididymis thus having insufficient time for epidydimal maturation [9, 71]. Boars collected twice a day for four consecutive days had more proximal droplets, more head and tail abnormalities and lower motility than control boars collected once every other day in the same period [9]. After 4 days of collection, the motility in the ejaculates of boars submitted to high frequency collection was not higher than 20%. The authors of the latter study suggested that high collection frequency resulted in an imbalance in the secretion of fluids in the epididymis which is necessary for sperm maturation.

## Bacterial contamination

Bacterial contamination is known to be detrimental to semen quality since it will cause sperm agglutination and will reduce motility [67]. It may also decrease the longevity of the sperm during storage and its fertility potential [69, 70, 72]. Commonly isolated bacteria from extended semen and their effect on sperm quality are summarised in Table 2.

**Table 2:** Percentage of contaminated extended semen samples^a^ in which different bacteria were isolated and their effect on sperm quality

Bacteria	Althouse and Lu. [67]	Schulze et al., [13]	Úbeda et al. [28]	Effect on sperm
% of conatminated samples (n/total samples)	31.2% (78/250)	25.6% (88/344)	14.7% (263/1785)	Reduced sperm quality
*Achromobacter xylosoxidans*	10.3%	3.4%	ND	Agglutination, poor motility, damaged acrosomes, acidic pH [44]
*Burkholderia cepacia*	2.6%	ND	ND	Agglutination, poor motility, damaged acrosomes, acidic pH [44]
*Clostridium perfringens*	ND	ND	ND	Poor sperm viability and motility [72]
*Enterobacter cloacae*	2.6%	13.6%	ND	Agglutination, poor motility, damaged acrosomes, acidic pH, decreased the osmotic resistance [44; 74]
*Enterococcus spp.*	20.5%	8%	ND	ND
*Escherichia coli*	6.4%	ND	1.5%	Agglutination, poor motility, damaged acrosomes [44]
*Klebsiella spp.*	3.8%	8%	11.8%	Poor motility [28]
*Leifsonia aquatic*	ND	20.5%	ND	ND
*Morganella morganii*	ND	ND	3.8%	Poor motility, damage acrosome, poorer response to the hypoosmoic swelling test [28]
*Proteus mirabilis*	1.3%	5.7%	1.9%	poor motility, abnormal forms [28]
*Providencia spp.*	3.8%	ND	9.1%	ND
*Pseudomonas spp.*	6.4%	5.7%	ND	*P. aureaginosa* reduced total and progressive sperm motility, sperm viability and acrosome integrity
*Ralstonia pickettii*	ND	11.4%	ND	ND
*Stenotrophomonas maltophilia*	15.4%	ND	ND	Agglutination, poor motility, damaged acrosomes
*Serratia marcescens*	10.3%	2.3%	12.5%	Agglutination, poor motility, damaged acrosomes, acidic pH [44]

^a^The percentage refers to the total contaminated samples. Bacteria present in a percentage of samples lower than 5% and identified only in one of the studies and for which no effect on sperm quality has been described, are not included in the Table

*ND* not described

The effect on sperm refers also to studies where semen was challenged with the different pathogens

Many different bacteria have been isolated from both raw semen and from extended semen doses [16, 44]. They mostly belong to the Enterobacteriaceae family [67, 73]. Within this family, *Serratia marcenses*, *Klebsiella oxytoca*, *Morganella morganii*, or *Proteus mirabilis* have been demonstrated to be present in a high percentage of samples and their presence is associated with reduced motility [73]. *Enterobacter cloacae* at a sperm:bacteria ratio of 1:5 and 1:10 reduced sperm motility and membrane integrity and resulted in sperm agglutination in semen doses stored at 15–17 °C [74]. *Clostridium perfringens* reduced sperm motility and viability after inoculation of 10^8^ cfu/ml into semen doses and 24 h incubation at 37° or storage at 15 °C [72]. Similarly, experimental contamination with 2 × 10^7^ or 2 × 10^8^ cfu/mL of *Pseudomonas aeruginosa* of stored boar semen resulted in a significant decreases in the percentages of total and progressive sperm motility, sperm viability and acrosome integrity, but did not affect pH [70]. In a study by Althouse et al. [44], 80 mL (3.5 x l0^9^ total sperm/dose) extended semen samples were inoculated with 10 to 15 colonies of pure cultures of the six most frequently isolated bacteria (*Enterobacter cloacae, Escherichia coli, Serratia marcescens, Alcaligenes xylosoxidans, Burkholderia cepacia, Stenotrophomonas maltophilia*). For all isolates, visual clumping, microscopic sperm to sperm agglutination (>25% of sperm), poor motility and damaged acrosomes were observed after inoculation in a time dependent manner. The exact interactions of the different bacteria with sperm are not yet fully investigated. Very recently, it has been suggested that *Pseudomonas aeruginosa* decreases the ability of sperm to accomplish capacitation [75]. In the latter study, it was found that inoculating sperm with 10^6^ or 10^8^ cfu/mL of *Pseudomonas aeruginosa* in capacitation media*,* results in more sperm with membrane damage and in a reduction in sperm motility kinetic as well as a decrease in phosphotyrosine levels of p32, the latter being a known marker of in vitro capacitation achievement. Risk factors for bacterial contamination during collection include preputial liquid trickling from the hand of the technician and collection longer than 7 min [68]. The CFU counts were also higher when boars had long preputial hair. Further, hygienic critical control points in semen processing in the laboratory have also been investigated [13]. It was found that species cultured from the contaminated extended semen were different from those isolated from the raw ejaculate, indicating that a great part of the semen dose contamination originates from the laboratory environment and not from the boar. Sinks or drains showed high contamination and multiple multidrug resistant bacteria were isolated in the different points of production. Controlling and assuring good hygiene practices in these critical points will result in lower bacterial counts and better sperm quality [16].

## Managing the ejaculate: Factors during semen handling

Different factors that have an effect on semen quality during semen handling have been investigated in recent years and are summarised in Table 3. Implementing quality control and quality assurance systems in AI centres would help diminishing the negative impact of these factors on semen quality.

**Table 3:** Semen handling factors affecting boar semen quality

Factor	Details	Effect of semen quality	Reference
Collection	Latex gloves.	Toxic for the sperm	[76]
	Preputial liquid into the collection container	Increases bacteriospermia	[68]
	Collection longer than 7 min,	Increases bacteriospermia	[68]
Dilution			
Temperature	Final dilution at 22 °C vs 30 °C (2-step dilution)	No differences in sperm motility, morphology or acrosome integrity after 3 days storage	[14]
	Final dilution at 20 °C vs 32 °C (2-step dilution)	No differences on membrane integrity or responsiveness to capacitating conditions	[82]
	Final dilution at 21 °C vs 32 °C (2-step dilution)	Lower motility, increased membrane damage after 6 days storage	[80]
Dilution rate	0.5 × 10^9^ sperm/80 ml vs 2.5 × 10^9^/80 ml	Lower motility	[83]
Storage media	Short vs long	Lower motility after 4 days storage	[15]^a^
	Magnetized extender	Improve membrane integrity	[88]
	Antibiotics	Prevent bacterial overgrowth	[92]
Packaging	Bags vs tubes.	Need less time to reach 17 °C	[96]
	Some plastic compounds	Toxic for sperm	[97]
Storage			
Temperature	< 12 °C	Reduced sperm motility and vitality	[78]
Duration	> 4 days	Reduced motility and fertility	[15, 86]
Air contact	Air contact during storage	Increase in pH, reduced sperm motility	[101]
Turning doses	180° rotation/12 h or five 360° rotations/h vs. non-rotated tubes, using	Increase in pH, reduced sperm motility	[102]

^a^Effect only seen in 1 of the 3 investigated long term extenders compared to 2 short term extenders

### Semen collection

Semen collection in AI centres is normally performed by the gloved handed technique [1]. Polyvinyl gloves can be used; latex gloves should be avoided as these are toxic for the sperm [76]. Rapid cooling could cause damage to the sperm and therefore a pre-warmed (38 °C) collection container is used [77]. Moreover, semen collection has been identified as the most critical point for bacterial contamination [68]. The first part of the ejaculate (~25 ml) should be discarded because it does not contain sperm and it may have a high bacterial count [68]. Subsequently the sperm-rich fraction is collected (40–100 mL) which contains 80–90% of all sperm cells in the ejaculate. Once the sperm-rich fraction is entirely collected the remainder of the ejaculate is again a clearer, watery fluid which need not be collected as it contains few sperm and is mainly secretions of the vesicular, prostate, and, towards the end of the ejaculation, bulbourethral glands.

### Dilution procedures

After ejaculation, sperm motility and vitality will only be retained for a few hours. To avoid early exhaustion and to prolong sperm survival, its metabolic activity must be decreased by chemical inhibitors and/or by lowering the temperature and, therefore, the ejaculate needs to be extended shortly after collection. Compared to semen of other animal species, boar sperm is very sensitive to temperatures below 12 °C due to a lower proportion of PUFA in its membrane [78, 79]. This temperature is normally not reached during semen processing where the temperature is controlled. The temperature of the ejaculate at the moment of collection is approximately 37 °C and is 32–35 °C upon arrival in the laboratory where it is processed [80]. Most AI centres use a two-step dilution in which semen is first diluted (1:1) with preheated extender (~33 °C) and subsequently diluted in either a preheated extender or an extender kept at room temperature [1, 80]. However, the dilution protocols and the temperature of extender for each dilution vary between AI centres [1]. It has been suggested that acclimation at 30 °C for several hours has a protective effect for samples to be stored at 17 °C [81]. However, Petrunkina et al. [82] showed a negative effect of acclimation at 32 °C compared to dilution at 20 °C, based on in vitro response to capacitation assays. They argued that, by keeping sperm closer to the physiological temperature, the sperm does not diminish its metabolism leading to changes that would otherwise impair semen quality. Lopez Rodriguez et al. [14] showed that when a 2-step dilution is performed, preheating the extender for the second dilution to match the semen temperature did not improve sperm motility, viability or acrosome integrity compared to a dilution at moderate room temperature (22–23 °C). In contrast, Schulze et al. [80] found lower motility and more membrane damage in sperm diluted at 21 °C compared to 32 °C in a 2-step dilution protocol. In the latter study however, most differences became only apparent after 6 days of storage, so it is likely of little commercial relaevance. When comparing both studies, it is worth mentioning that in the study of Lopez Rodriguez et al. [14], samples were investigated during only 3 days, which is a commonly used storage duration on farm. Although no apparent effect of dilution temperature on semen quality was found in vitro, in vivo studies elaborating on the effect of dilution temperature on fertility of boar semen are needed to confirm that sperm diluted at different temperatures do not lose their capability to fertilize ova.

Additionally, the dilution rate seems to have an effect on the quality of the sperm during storage. High dilution (0.5 × 10^9^ sperm/80 ml or 1 × 10^9^ sperm/80 ml) resulted in lower sperm motility during storage compared to a lower dilution (2.5 × 10^9^ sperm/80 mL) but addition of seminal plasma could alleviate this negative effect [83].

### Storage media

The media used for liquid storage are necessary to prolong sperm survival by providing energy to the cells, buffering the pH of the suspension and avoiding the growth of bacteria [15]. Many different boar semen extenders either for short or long-term storage are available claiming protection [15]. Long-term extenders contain more complex buffering systems (HEPES, Tris) in addition to the bicarbonate buffering system, and they also contain bovine serum albumin [78, 84]. The latter has a positive influence on sperm survival due to the absorption of metabolic bacterial products from the extender [85]. According to in vitro and in vivo studies, most extenders on the market provide an acceptable sperm vitality protection during the first 72 h of storage, although motility and fertility decrease when semen is stored during more than 4 days [15, 86].

Extender concentrates are normally diluted in distilled or de-ionized water. Not only is the microbiological quality of the water important but also the electrolyte content, especially the absence of calcium ions. Recently, magnetized extender has been shown to improve membrane integrity by reducing peroxidation [87, 88]. It was proposed that magnetising the water would increase the electron donor ability of the semen extender and this could decrease the levels of free radicals and reactive oxygen species. However, benefitial effects of magnetised water were only observed in samples stored for 120 h or 168 h, whereas semen doses are normally used within 72 h.

Hormone supplementation of AI doses has been suggested to improve fertility. Addition of oxytocin to AI doses improved farrowing rate during summer months in Spain [89]. Another study showed that addition of oestrogens, prostaglandin F2α (PGF2α) and oxytocin to AI doses did not improve pregnancy rate but increased the total number of foetuses [90]. No improvement of sperm motility was observed when PGF2α (2.5, 5 or 10 mg of dinoprostum) was added to 100 mL diluted porcine semen [91]. These hormones do not seem to have an effect on sperm quality and their effect on sow fertility are difficult to determine given the many factors contributing to reproductive outputs.

As bacterial contamination is present in extended boar semen, antibiotics are commonly added to prevent bacterial overgrowth and to reduce the effect of bacterial toxins [92]. In the context of prudent use of antimicrobials, less use of antimicrobials in diluted semen may help to reduce antimicrobial resistance. In this respect, single layer centrifugation of boar ejaculates can reduce bacteria concentration and consequently reduce the need for antibiotics in semen extenders [93]. Although this process does not seem to have an effect on total motility, it does appear to increase sperm motion linearity. Its effect on semen quality and sperm fertility deserves further investigation. Addition of a selected cyclic hexapeptide has been proposed as a replacement for antibiotics in extenders, but the potential of these peptides is still under investigation [94].

### Packaging and storage

After dilution is completed, diluted semen is packaged in 80–100 mL doses to be stored and distributed. Doses normally contain 2–3 billion sperm. However, during recent years, new techniques (e.g. intra-uterine insemination) have been developed to allow insemination of lower numbers of sperm in a smaller volume [95].

The packaging process is done by automated systems in most AI centres. These systems are fast and accurate and may not damage sperm. Different containers such as plastic bottles, blisters, tubes or a collapsible membrane with an integrated catheter can be used for storage, delivery and insemination of extended semen doses [1]. It has been shown that the type of container will influence cooling rate and it seems that bags need less time to reach 17 °C compared to tubes [96]. Also, the plastic compounds in the different packages should be investigated in cases of reduced sperm quality, since they may be toxic for sperm [97]. The latter study was the first report linking reproductive failure in sows to reduce sperm quality caused by to the presence of cyclic lactone and bisphenol A diglycidyl ether (BADGE) in the semen packages. They confirmed this association in vivo by adding those two chemicals to semen and inseminating two groups of 50 sows with either the mixture or a control. The addition of cyclic lactone and BADGE was associated with a reduction in fertility (58% vs 84% in the control sows). Interestingly, the addition of these chemicals to the semen did not cause sperm damage that could be observed with the routine sperm quality analyses such as membrane functionality, abnormal morphology, concentration, sperm motility and acrosome status. Analyses for related toxins should therefore be included in the routine quality control of companies delivering semen for AI.

Sperm can be encapsulated in barium alginate, protecting the sperm from damage during handling. The concentration in each capsule is ejaculate dependent and they are inseminated in a conventional way giving good fertility results [98]. Thus, this process seems not to impair semen quality. Sperm encapsulation has, however, not reached commercial application likely due to the higher cost compared to extended semen [99].

Further storage of diluted semen is performed at 17 °C. At this temperature semen metabolism is reduced, a condition necessary to extend the storage time [78]. The critical lower temperature for sperm survival in pigs was established at 12 °C whereas storage at 15–17° showed no detrimental effect on boar sperm motility and vitality [78]. The mechanism behind the aging of sperm during storage has been studied by means of new semen quality assays. The results showed that it is related, among other factors, to lipid peroxidation [100] and changes in the fluidity of the sperm membrane initiating capacitation like changes [82].

Air contact during storage should be avoided, as it increases the pH which is negatively correlated with sperm motility [101]. Therefore, many different buffering systems are used to stabilize the pH.

Very recently, Schulze et al. [102] showed that turning doses during storage to avoid sedimentation has a detrimental effect on sperm motility. Although the biological mechanism could not be explained, the authors hypothesized that this was due to increased oxidative stress.

The hypothesis that controlled stress before storage may protect sperm has also been investigated. A study involving 7 hybrid boars has shown that stressing semen with hydrostatic pressure compared to conventional processing resulted in higher progressive motility [103]. In a second part of the latter study, each ejaculate of 14 hybrid boars was split in two and either treated by hydrostatic pressure or processed normally. Subsequently, 104 females were inseminated with either treated or control semen and an increased litter size was observed in gilts inseminated with the stressed semen but interestingly, this effect was not seen in multiparous sows [103].

## Conclusions and future perspectives

This literature review shows that boar management and semen handling in AI centres can be improved. Genes associated with semen quality could be used for early detection of boars with higher fertility potential. Boar housing and climate seem only to affect semen quality when boars are submitted to extreme conditions. Feeding seems to have an effect on boar semen quality only when there are severe deficiencies in the diet. Nonetheless, recent research has shown that some feeding strategies may improve resistance of sperm to storage. Unfortunately, infertility in boars and how to treat the condition is only scarcely documented in literature and is mainly diagnosed at the slaughterhouse. Once the boars are in production, early detection of boars that are no longer able to deliver good semen is still a challenge. Bacterial contamination affects semen quality and further research in antimicrobial resistance and on how to reduce contamination is warranted. Semen processing is not yet standardized among AI centres and the critical points during production need to be identified. Detailed studies on temperature effects at each step of semen handling may help to improve and eventually simplify the currently used semen production systems. The different packing systems might have an effect on semen quality and they must also be investigated when reduced semen quality in the AI doses is observed. Altogether, AI centres would benefit from the implementation of standardised quality control and quality assurance systems.

## Abbreviations

AI: Artificial insemination
BADGE: Bisphenol A diglycidyl ether
CASA: Computer assisted semen analysis
CFU: Colony-forming units
DNA: Deoxyribonucleic Acid
GnRH: Gonadotropin-releasing Hormone
GPx: Glutathione peroxidase
PCV2: Porcine circovirus type 2
PGF2α: Prostaglandin F2α
PRRSv: Porcine reproductive and respiratory syndrome virus
PUFA: Polyunsaturated fatty acid
